# Two Different Antibody-Dependent Enhancement (ADE) Risks for SARS-CoV-2 Antibodies

**DOI:** 10.3389/fimmu.2021.640093

**Published:** 2021-02-24

**Authors:** Darrell O. Ricke

**Affiliations:** Biological and Chemical Technologies, Massachusetts Institute of Technology Lincoln Laboratory, Biotechnology and Human Systems, Lexington, MA, United States

**Keywords:** antibody dependent enhancement, ADE, COVID-19, SARS-CoV-2, multisystem inflammatory syndrome, MIS-C, antibody dependent enhancement

## Abstract

COVID-19 (SARS-CoV-2) disease severity and stages varies from asymptomatic, mild flu-like symptoms, moderate, severe, critical, and chronic disease. COVID-19 disease progression include lymphopenia, elevated proinflammatory cytokines and chemokines, accumulation of macrophages and neutrophils in lungs, immune dysregulation, cytokine storms, acute respiratory distress syndrome (ARDS), etc. Development of vaccines to severe acute respiratory syndrome (SARS), Middle East Respiratory Syndrome coronavirus (MERS-CoV), and other coronavirus has been difficult to create due to vaccine induced enhanced disease responses in animal models. Multiple betacoronaviruses including SARS-CoV-2 and SARS-CoV-1 expand cellular tropism by infecting some phagocytic cells (immature macrophages and dendritic cells) via antibody bound Fc receptor uptake of virus. Antibody-dependent enhancement (ADE) may be involved in the clinical observation of increased severity of symptoms associated with early high levels of SARS-CoV-2 antibodies in patients. Infants with multisystem inflammatory syndrome in children (MIS-C) associated with COVID-19 may also have ADE caused by maternally acquired SARS-CoV-2 antibodies bound to mast cells. ADE risks associated with SARS-CoV-2 has implications for COVID-19 and MIS-C treatments, B-cell vaccines, SARS-CoV-2 antibody therapy, and convalescent plasma therapy for patients. SARS-CoV-2 antibodies bound to mast cells may be involved in MIS-C and multisystem inflammatory syndrome in adults (MIS-A) following initial COVID-19 infection. SARS-CoV-2 antibodies bound to Fc receptors on macrophages and mast cells may represent two different mechanisms for ADE in patients. These two different ADE risks have possible implications for SARS-CoV-2 B-cell vaccines for subsets of populations based on age, cross-reactive antibodies, variabilities in antibody levels over time, and pregnancy. These models place increased emphasis on the importance of developing safe SARS-CoV-2 T cell vaccines that are not dependent upon antibodies.

## Introduction

The SARS-CoV-2 virus is a unclassified betacoronavirus with sequenced genomes ranging from 29.8 to 29.9 k RNA bases. The SARS-CoV-2 genome encodes replicase proteins, structural proteins, and accessory proteins (1). The ORF1a and ORF1ab polyproteins are proteolytically cleaved into 16 non-structural proteins designated nsp1-16 (1). Like SARS, COVID-19 manifests as a virulent zoonotic virus in humans with currently 101,211,750 global cases and 2,183,169 deaths as of Jan. 28, 2021 (2). The details of SARS-CoV-2 infections and disease progression are still being worked out. One proposed step in COVID-19 disease progression involves the nucleocapsid protein binding to the prostaglandin-endoperoxide synthase 2 (PTGS2)/cyclooxygenase-2 (COX-2) promoter and upregulating expression resulting in elevated levels of prostaglandin E_2_ (PGE_2_) and other inflammatory molecules (3–5). Elevated PGE_2_ may be driving hyper-activation of mast cells associated with excess release of histamine and additional inflammatory molecules (5). COVID-19 is predicted to be a mast cell disease (6).

Zoonotic MERS-CoV, SARS-CoV-1, and SARS-CoV-2 are evolutionarily related with similarities in disease progression in humans. The mild variant first phase of viral progression generally presents with mild flu-like symptoms. For some individuals, infection progresses to a second moderate-severe variant phase. Progression to this phase coincidently coincides with timing of anticipated humoral immunity antibody response from memory B-cells for cross reactive antibodies. Coronavirus infection of phagocytic cells has been previously observed. MERS-CoV can infect monocyte-derived macrophages (MDMs), monocyte-derived dendric cells (MoDCs), and T cells (7, 8). In a mouse animal model, phagocytic cells contribute to the antibody-mediate elimination of SARS-CoV-1 (9). This process is expected for patients with mild symptoms who do not progress to moderate or severe disease. For patients with moderate and severe symptoms, pathophysiology is consistent with infection of phagocytic immune cells (immature MDMs and MoDCs). Chemokines attract additional dendritic cells and immature macrophages that are susceptible to infection leading to a possible infection amplifying cascade of phagocytic immune cells. For some patients with severe symptoms, excessive accumulation of macrophages contributes toward a storm of cytokines (10–12) and chemokines. These viruses also perturb the adaptive immune responses within infected individuals. Individuals with SARS have pronounced peripheral T cell lymphocytopenia with reduced CD4^+^ and CD8^+^ T cells (13, 14). MERS-CoV and SARS-CoV are associated with T cell apoptosis (15, 16). Infection of macrophages (17) and some T cells along with viral dysregulation of cellular pathways result in compromised innate and humoral immunity in patients in phase II (18). The possibility of migration throughput the body of infected immune cells and later high virus titer in blood can account for additional disease pathophysiology clinical observations observed for these viruses. Other disease differences may simply be the different population of cells with target host receptors angiotensin I converting enzyme 2 (ACE2) for SARS-CoV-1 and SARS-CoV-2 and dipeptidyl peptidase IV (DPP4) for MERS-CoV. The increased affinity of the SARS-CoV-2 Spike protein receptor-binding-domain (RBD) compared to SARS may account for the significant airborne transmission of SARS-CoV-2 (19). Also, neuropilin-1 facilitates SARS-CoV-2 cell entry and infectivity (20).

Characterizing variability of viral proteins can inform designing medical countermeasures (MCMs). For viral progeny, deleterious mutations are selected against (21). Neutral mutations (22) provide a framework for antigenic drift to facilitate escape from immune responses; these residues will continue to mutate over time. The critical-spacer model proposes that proteins have either amino acid residue side-chains critical for function or have variable side-chains while possibly function for positioning/folding of critical residues (23). The divergence model of protein evolution proposes that number of critical residues for a protein is consistent for evolutionarily closely related proteins (24). These concepts are applied to SARS-CoV-2 Spike (S) protein leveraging closely related coronavirus protein sequences to provide insights into viral vulnerabilities that can be leveraged in designing MCMs. The exposed domain of the Spike protein exhibits exposed surface areas with high variability. Increased risk for antibody-dependent enhancement (ADE) from antibodies targeting SARS-CoV-2, SARS-CoV-1, and MERS-CoV exposed residues is indicated by observed ADE in animal models and the antibody facilitated infection of phagocytic immune cells by coronaviruses (9, 25). In addition, SARS-CoV-2 antibodies bound to mast cells may also be involved in ADE for some MIS-C and MIS-A patients (26).

## Methods

SARS-CoV-2 spike protein sequence from GenBank entry MN908947.3 was searched against the non-redundant (nr) and PDB database using the NCBI BLASTP web interface. Hit protein sequences were downloaded. Protein multiple sequence alignments were created with the Dawn program (27). The Spike structure 6CRZ (28) was downloaded from RCSB PDB database (29). Dawn variation results were visualized with the Chimera program (30).

## Results

**Figure 1:**
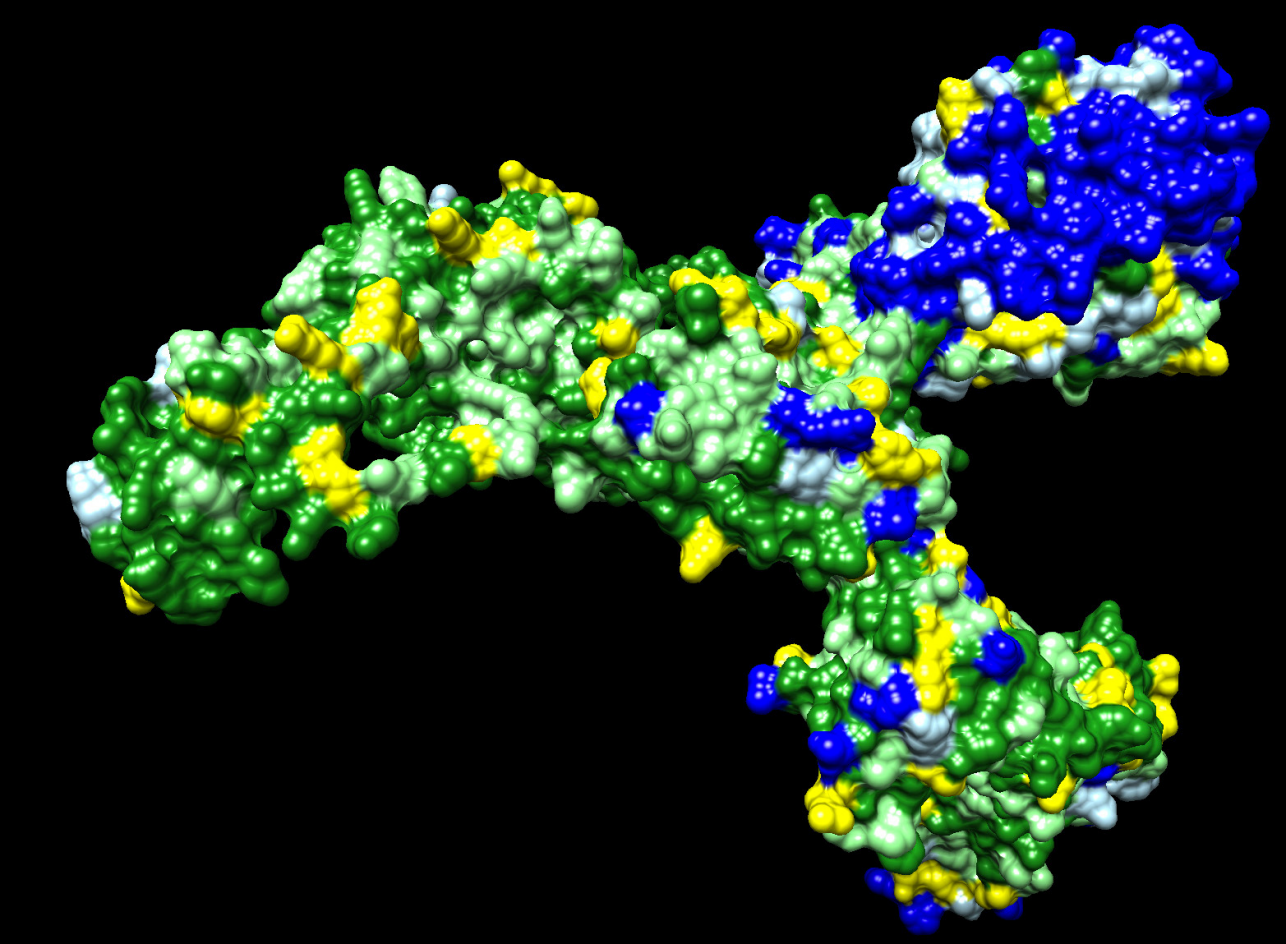
SARS-CoV-2 Spike protein variation results. Amino acid residue color code: dark green (critical residues—V1), light green (critical residues with conservative substitutions or variant in <10 sequences—V2, yellow (three variants—V3), light blue (four variants—V4; likely spacer residues), and blue (5+ variants—V5+; spacer residues).

Dawn variation (V <n>) results for SARS-CoV-2 amino acid residues were classified as 650 V1 residues—dark green, 263 V2 residues—light green, 123 V3 residues—yellow, 107 V4 residues—light blue, and 152 V5+ residues—dark blue (Figure 1). The dark green residues represent candidate critical residues and the dark blue residues represent candidate spacer residues (Figure 1). Amino acid residues with conservative substitutions are also consider critical residues, and are colored light green in Figure 1; positions with > 95% of a single residue were included in this category to accommodate potential sequencing errors and possibly adaptative mutations. The V1+V2 residues represent 71% of the 1,295 Spike residues. The Spike protein exhibits regions of extensive variability of exposed surface residues (Figure 1).

## Discussion

### Variation Results

The observed amino acid variations in SARS-CoV-2 proteins are consistent with expected natural variations in the context of random mutations and selection in the context of host immune responses. The Spike protein S1 extended domain shows the highest number of exposed surface highly variable residues (Figure 1). These spacer residues may function as exposed antigens for antibody responses with the possibility of suppressing immune responses to less immunogenic surface antigens. Many of these Spike protein antigens may lead to non-neutralizing antibodies. Mutations at these residues may provide antigenic drift to escape immune responses. As the COVID-19 pandemic continues, Spike mutation variants are accumulating resulting in the design of vaccine booster shots prior to initial population vaccinations (31). The Spike protein represents an evolving vaccine target with parallels to the annual influenza vaccine hemagglutinin and neuraminidase targets while the COVID-19 pandemic persists enabling rapid virus evolution in humans.

### Multiple Coronaviruses Approaches for Cell Infection

Coronaviruses have multiple approaches for infecting cells by direct receptor binding and by indirect antibody Fc uptake. The SARS-CoV-2 Spike protein contains receptor-binding domains (RBD) targeting human angiotensin I converting enzyme 2 (ACE2) (32, 33); this is the initial route for infecting host cells. To take advantage of antibody responses, coronaviruses also leverage antibody Fc uptake to infect some phagocytic immune cells (34). Coronaviruses use the Spike protein subunit 2 fusion peptide (FP), heptad repeat 1 (HR1), and heptad repeat 2 (HR2) to infect immune cells upon proteolytic cleavage of Spike within endosomes. HR1 and HR2 form a canonical 6-helix bundle involved in membrane fusion (35). Jaume et al. (34) found that antibody-mediated infection was dependent on Fc receptor II and not the endosomal/lysosomal pathway utilized by ACE2 targeting. Viral infection of complement receptor (CR) cells is an additional possible route of infecting cells expanding cellular tropism (36). This expanded cellular tropism mechanism provides coronaviruses like SARS-CoV-1, MERS-CoV, and SARS-CoV-2 with more than one cellular trophism for infecting host cells. This leads to the prediction that antibody mediated uptake of virus is the potential mechanism that induces ADE to cross-reactivity antibodies, maternally transferred antibodies (matAbs), and vaccines (37–40).

### Macrophages and Immune Dysregulation

**Figure 2:**
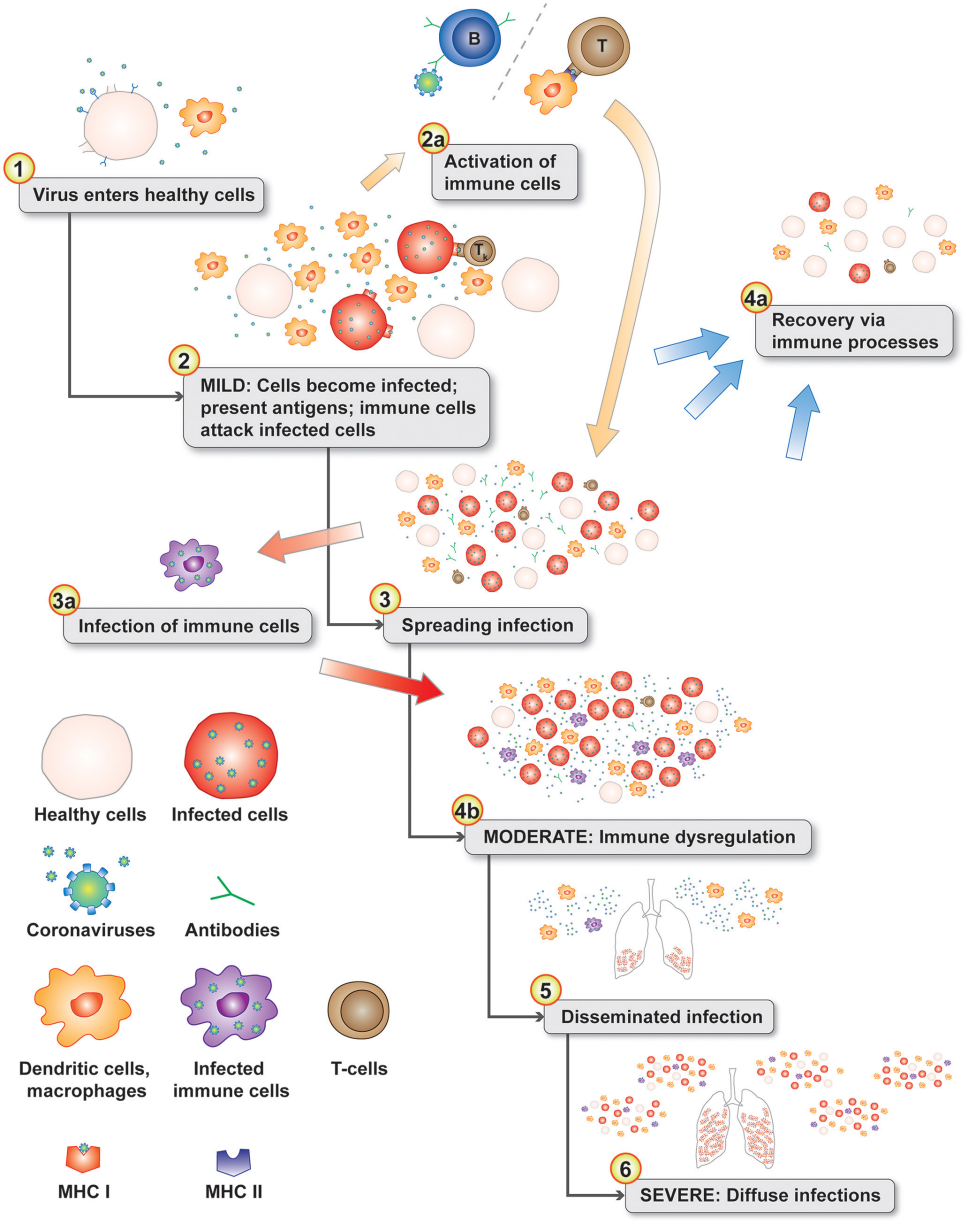
Disease progression model with normal immune responses during the initial mild symptoms phase (see 1–3). Antigen presenting cells migrate to the lymph nodes to activate T cells (2a). The progression gate to moderate and server disease is the infection of phagocytic immune cells (3a) leading to immune dysregulation (4b). In the lungs, chemokines attract additional dendritic cells and immature macrophages that are subsequently infected in an positive feedback-loop infection cascade (4b). Infected phagocytic immune cells disseminate throughout the body infecting additional organs (5 & 6). Levels of chemokine and cytokines in the lungs from infected cells can create a cytokine storm (6).

Macrophages play an important role in disease progression and possibly immune dysregulation for SARS and COVID-19. Lymphopenia is a common feature in patients with SARS (13, 41) and COVID-19 (42, 43). Direct infection of subpopulations of immune cells is possible if they express virus target receptors. Two receptors have been identified for SARS-CoV-1 including ACE2 (44) and C-type lectin domain family 4 member M (CLEC4M, CD209L, CD299, DC-SIGN2, DC-SIGNR, HP10347, and LSIGN) (45) with CLEC4M expressed in human lymph nodes (46). In a mouse model, depletion of CD4+ T cells resulted in an enhanced immune-mediated interstitial pneumonitis when challenged with SARS-CoV-1 (47). But, depletion of CD4+ and CD8+ T cells and antibodies enabled the innate defense mechanisms to control the SARS-CoV-1 virus without immune dysregulation (47). Similar results were also observed in mice with SARS-CoV-1 challenge, but treatment with liposomes containing clodronate, which deplete alveolar macrophages (AM), prevented immune deficient virus-specific T cell response (48). These studies point to an interplay between antibodies and macrophages in ADE responses in animal models. In a macaque model, anti-spike IgG causes acute lung injury by skewing macrophage response toward proinflammatory monocyte/macrophage recruitment and accumulation during acute SARS-CoV-1 infection (49). Blockade of *in vitro* human activated macrophages FcγR reduced proinflammatory cytokine production (49). CD169+ macrophages have ACE2 and are susceptible to SARS-CoV-2 infection (50). Both M1- and M2-type macrophages are susceptible to SARS-CoV-2 infection (51). These observations are likely linked by antibody-dependent enhancement of coronavirus infection of macrophages (34, 52). The pathophysiology of moderate and severe SARS and COVID-19 diseases fits a proposed model of antibody-dependent infection of macrophages as the key gate step in disease progression from mild to moderate and severe symptoms contributing to dysregulated immune responses (53) including apoptosis for some T cells/T cell lymphopenia, proinflammatory cascade with macrophage accumulation, and cytokine and chemokine accumulations in lungs with a cytokine storm in some patients. Infected phagocytic immune cells may enable the virus to spread to additional organs prior to viral sepsis (Figure 2).

### Antibody-Dependent Enhancement (ADE) of Coronaviruses

Antibody-dependent enhancement (ADE) may develop via more than one molecular mechanism. One model suggestions that antibody/Fc-receptor complex functionally mimics viral receptor enabling expanded host cell trophism of some phagocytic cells (54). Wan et al. (54) illustrate an antibody dosage effect for enhancing disease or inhibiting the virus dependent upon the antibody dosage. It is well-established that antibodies to one strain of a virus may be subneutralizing or non-neutralizing for viral infections of different strains (55–57). Infection of cells expressing Fc-gamma was shown for SARS-CoV-1 (58). A possible case of ADE was observed in a patient with a second SARS-CoV-2 infection (59). Early vaccine results show significant antibody responses by day 14 (60) which represents memory B-cell responses (i.e., original antigenic sin) with cross-reactivity antibodies from likely other coronavirus strain(s). Early high antibody responses are correlated with increased disease severity for both SARS (61) and COVID-19 (62–67). Wu et al. demonstrated that antibodies from COVID-19 patients enabled SARS-CoV-2 infections of Raji cells (lymphoma cells derived from B lymphocytes), K562 cells (derived from monocytes), and primary B cells (68). SARS-CoV-2 infection of some phagocytic cells (i.e., macrophages) may be a key gate step in disease progression for some patients.

### Mast Cells Risks for ADE and Multisystem Inflammatory Syndromes (MIS-C & MIS-A)

Mast cells can degranulated by both IgE and IgG antibodies bound to Fc receptors (69). Cardiac injury is a common condition among hospitalized COVID-19 patients and is associated with higher risk of mortality (70). However, pathological manifestations of heart tissues found only scarce interstitial mononuclear inflammatory infiltrates without substantial myocardial damage (42). Myocardial injury significantly correlates with fatal outcome for COVID-19 (71). Multisystem inflammatory syndrome in children (MIS-C) and adults (MIS-A) associated with COVID-19 has appeared in areas following SARS-CoV-2 outbreaks. A model of MIS-C has been proposed where activation and degranulation of mast cells with Fc receptor-bound SARS-CoV-2 antibodies leads to increased histamine levels (26). This model is consistent with MIS-C in infants with maternally transferred antibodies (matAbs) (37–40) to SARS-CoV-2. SARS-CoV-2 nucleocapsid binding to PTGS2 prompter resulting in upregulated prostaglandin E2 (PGE_2_) in COVID-19 patients (4). Elevated PGE_2_ may be driving hyper-activated mast cells as an alternative mechanism driving increased histamine levels in older children and adults. These increased histamine levels are predicted to impede blood flow through cardiac capillaries due to constricted pericytes with increased risk for cardiac pathology due to cell death by anoxia and coronary artery aneurysms due to increased blood pressure (26). An instance of a 12 years old child with a previous asymptomatic COVID-19 infection developing MIS-C on likely second infection has been reported (72).

### Vaccine Risks for Antibody-Dependent Enhancement (ADE)

Virus vaccines can use live-attenuated virus strains, inactivated (killed) virus, protein subunit, messenger ribonucleic acid (mRNA), or deoxyribonucleic acid (DNA) vaccine. Antibodies induced by vaccines can be neutralizing or non-neutralizing. Non-neutralizing antibodies can contribute to anti-viral activities with mechanisms including antibody-medicated complement-dependent cytotoxicity (CDC), antibody-dependent cellular cytotoxicity (ADCC), antibody-dependent cellular phagocytosis (ADCP) [reviewed (73)]. The yearly influenza vaccine induces both neutralizing and non-neutralizing antibodies that provide projection against the strains in the vaccine and closely related strains. Vaccine-associated enhanced disease (VAED) can result when there are multiple circularizing serotypes of virus [e.g., Dengue fever (55–57)] or when the virus uses antibodies for expanded host cell trophism of phagocytic immune cells.

Many of the viruses associated with ADE have cell membrane fusion mechanisms (38). For influenza A H1N1, vaccine-induced cross-reactive anti-HA2 antibodies in a swine model promote virus fusion causing vaccine-associated enhanced respiratory disease (VAERD) (74). ADE was observed for the respiratory syncytial virus (RSV) in the Bonnet monkey model (37). Van Erp et al. (37) recommends avoidance of induction of respiratory syncytial virus (RSV) non-neutralizing antibodies or subneutralizing antibodies to avoid ADE. ADE has been observed in multiple SARS-CoV-1 animal models. In a mouse model, attempts to create vaccines for SARS-CoV-1 lead to pulmonary immunopathology upon challenge with SARS-CoV-1 (75, 76); these vaccines included inactivated whole viruses, inactivated viruses with adjuvant, and a recombinant DNA spike (S) protein vaccine in a virus-like particle (VLP) vaccine. Severe pneumonia was observed in mice vaccinated with nucleocapsid protein after challenge with SARS-CoV-1 (77). Enhanced hepatitis was observed in a ferret model with a vaccine with recombinant modified vaccinia virus Ankara (rMVA) expressing the SARS-CoV-1 Spike protein (78). ADE was observed for rhesus macaques with SARS-CoV-1 vaccine (79). SARS-CoV-1 ADE is mediated by spike protein antibodies (80). Antibodies to the SARS-CoV-1 spike protein can mediate viral entry via Fc receptor-expressing cells in a dose-dependent manner (54). Jaume et al. (34) point out the potential pitfalls associated with immunizations against SARS-CoV-1 Spike protein due to Fc mediate infection of immune cells. This leads to the prediction that new attempts to create either SARS-CoV-1 vaccines, MERS-CoV vaccines (81), or SARS-CoV-2 vaccines have potentially higher risks for inducing ADE in humans facilitated by antibody infection of phagocytic immune cells. This potential ADE risk is independent of the vaccine technology (82) or targeting strategy selected due to predicted phagocytic immune cell infections upon antibody uptake. For MERS patients, the seroconversion rate increased with disease severity (83). Severe clinical worsening for SARS patients occurs concurrently with timing of IgG seroconversion (84). Clinical evidence of early high IgG responses in SARS patients is correlated with disease progression (85) and severity (62–67). Antibody treatments for critically ill COVID-19 patients have been halted due to a potential safety signal and unfavorable risk-benefit profile (86). Current SARS-CoV-2 vaccines appear to be providing protection with high antibody titers; the possibility of ADE risks associated with waning titers of antibodies over time remains unknown.

### Convalescent Plasma Therapy

Convalescent plasma therapy takes the antibodies from a recovering patient and provides them to patients with active infections. COVID-19 results for convalescent plasma therapy appear to have mixed results with no statistically significant improvement in randomized clinical trials (87, 88): in a trial, no significant difference in 28-days mortality (15.7 vs 24.0% odds ratio: 0.59, *p* = 0.30) was observed in a randomized trial (87); and, in the PLACID trial, progression to severe disease or all-cause mortality at 28 days occurred in 44 (19%) convalescent plasma arm vs. 41 (18%) control arm (risk ratio 1.04) (88). Neither trial mentions antibody-dependent enhancement in context of progression to severe disease or all-cause mortality. For SARS, a higher discharge rate was observed amount patients who were given convalescent plasma before day 14 of illness (58.3%) vs. after 14 days (15.6%), *p* < 0.001; the mortality rate for the second group was 21.9% which was higher than the all SARS-related mortality rate in Hong Kong of 17% (89); while this looks promising for most patients, the increased mortality above the regional average observed for patients after 14 days of illness should be noted.

### Antibody Targets

Analyzing the Cryo-EM structures of MERS-CoV and SARS-CoV-1 spike (S) glycoproteins, Yuan et al. (90) suggest that the fusion peptide (FP) and the heptad repeat 1 region (HR1) are potential targets for eliciting broadly neutralizing antibodies based on exposure on the surface of the stem region, with no N-linked glycosylation sites in this region, and sequence conservation. Antibodies that interrupt virus-cell fusion will likely block the infection of immune cells using Fc-mediated uptake of virus (34). This has been demonstrated for SARS-CoV-1 for antibodies to the HR2 region (91–93). Likewise, SARS-CoV-2 antibodies that block cell fusion are likely to not share the same ADE risk of other SARS-CoV-2 antibodies.

### B Cell Vaccine Designs

B cell vaccines that target the Spike protein cell fusion mechanisms have the highest chance of raising neutralizing antibodies with minimal or no ADE risk due to antibody binding sterically blocking cell fusion. Antibodies targeting other portions of the Spike protein or other SARS-CoV-2 exposed proteins may enable infection of phagocytic immune cells even if they are neutralizing.

### T Cell Vaccine Designs

T cell vaccines that target SARS-CoV-2 replicase proteins have the highest change of avoiding viral escape by antigenic variation and accumulation of mutations in variable residues. Lisziewicz and Lori (94) described an approach for developing a T cell COVID-19 vaccine. EpiVax EPV-CoV19 (95) is an example COVID-19 T cell vaccine.

## Summary

Given past data on multiple SARS-CoV-1 and MERS-CoV vaccine efforts have failed due to ADE in animal models (75, 81), it is reasonable to hypothesize a similar ADE risk for SARS-CoV-2 antibodies and vaccines. ADE risks may be associated with antibody level (which can wane over time after vaccination) and also if the antibodies are derived from prior exposures to other coronaviruses. In addition, ADE with mast cells likely plays a role in MIS-C for infants and possibly older MIS-C and MIS-A patients. While expanded trophism of SARS-CoV-2 represents a possible ADE risk in the subset of COVID-19 patients with disease progression beyond the mild disease stage.

## Data Availability Statement

The datasets presented in this study can be found in online repositories. The names of the repository/repositories and accession number(s) can be found in the article/Supplementary Material.

## Author Contributions

DR conceived of the presented ideas, analyzed the data, and wrote the manuscript.

## Conflict of Interest

The author declares that the research was conducted in the absence of any commercial or financial relationships that could be construed as a potential conflict of interest.
